# Are Viral Vectors Any Good for RNAi Antiviral Therapy? †

**DOI:** 10.3390/v12101189

**Published:** 2020-10-20

**Authors:** Kenneth Lundstrom

**Affiliations:** PanTherapeutics, Route de Lavaux 49, CH1095 Lutry, Switzerland; lundstromkenneth@gmail.com

**Keywords:** gene silencing, RNAi, shRNA, miRNA, viral vector, viral replication, antiviral drugs, COVID-19

## Abstract

RNA interference (RNAi) represents a novel approach for alternative antiviral therapy. However, issues related to RNA delivery and stability have presented serious obstacles for obtaining good therapeutic efficacy. Viral vectors are capable of efficient delivery of RNAi as short interfering RNA (siRNA), short hairpin RNA (shRNA) and micro-RNA (miRNA). Efficacy in gene silencing for therapeutic applications against viral diseases has been demonstrated in various animal models. Rotavirus (RV) miR-7 can inhibit rotavirus replication by targeting the RV nonstructural protein 5. Viral gene silencing by targeting the RNAi pathway showed efficient suppression of hepatitis B virus replication by adeno-associated virus (AAV)-based delivery of RNAi hepatitis B virus (HBV) cassettes. Hepatitis C virus replication has been targeted by short hairpin RNA molecules expressed from lentivirus vectors. Potentially, RNAi-based approaches could be suitable for antiviral drugs against COVID-19.

## 1. Introduction

Although the first antiviral drug against herpes simplex virus, idoxuridine, has been on the market since 1963, not too many efficient antiviral drugs have been developed during the past 60 years. There is, however, an urgent need for novel antiviral drugs, particularly due to the current devastating COVID-19 pandemic [1]. Gene silencing, particularly the application of RNA interference (RNAi), has presented a novel alternative approach for drug development. Although originally discovered in plants and *Caenorabditis elegans* worms, the phenomenon was later confirmed to occur in higher organisms including humans [2]. The mechanism of gene silencing can take place either through mRNA degradation or suppression and can be executed by small interfering RNA (siRNA), short hairpin RNA (shRNA) and micro-RNA (miRNA) [3]. One attractive feature of RNAi relates to its reversible function, providing only transient therapeutic efficacy due to the sensitivity of RNA to degradation, which supports application for treatment of acute but not chronic diseases. However, the enthusiasm for RNAi technology has been hampered by stability issues, RNA being sensitive to degradation, and complications of efficient delivery. In this context, viral vectors have proven efficient as delivery vehicles and especially self-replicating RNA viral vectors can provide additional efficacy through high-level cytoplasmic RNA amplification [4]. The current Special Issue on RNAi for Antiviral Therapy consists of an overview on the application of viral vectors for RNAi-based antiviral therapy [5], miRNA-based inhibition of rotavirus replication [6], a review on the suppression of hepatitis B virus (HBV) replication [7], and lentivirus-based shRNA targeting hepatis C virus (HCV) replication [8]. Therefore, it is justified to briefly present the essential elements for RNAi therapy, which relate to bioinformatics as a tool for target selection, vector engineering to provide more efficient and safer viral vectors and delivery-related issues to provide both a high safety standard and therapeutic efficacy. The progress and problems related to viral vector-based RNAi antiviral therapy are also discussed here, and a summary of the recent findings described in the Special Issue is included.

## 2. Gene Silencing of Viral Replication—Progress and Problems

The important role of bioinformatics in RNAi-based antiviral drug development can be exemplified by targeting of the HIV transactivating response region [9], and information on experimentally tested siRNA/shRNA sequences [10]. Moreover, siRNAs targeting the conserved regions of HIV, HCV, influenza virus and SARS-CoV have been designed [11]. Although bioinformatics can provide an excellent approach for prediction and in silico testing of siRNA efficacy prior to in vitro and in vivo studies, the RNA structure might impose restrictions for RNA-based therapy against HCV due to the properties of the HCV genome from primary sequence to tertiary structure [12]. 

RNA delivery has been hampered not only by degradation issues but also by the challenges related to cellular uptake due to the negatively charged cellular bilayer and RNA itself [13]. To prevent RNA degradation, lipids, cell-penetrating peptides and extracellular vesicles have been used for RNA delivery, including siRNA molecules [14,15]. Alternatively, viral vectors such as adenovirus (Ad), adeno-associated virus (AAV), retroviruses, lentiviruses [5] and self-replicating rhabdoviruses and alphaviruses [4] have been applied. However, viral vectors have their shortcomings. For instance, AAV administration has been associated with pre-existing immunity, leading to short-lived transgene expression after AAV re-administration. To address this problem, different AAV serotypes have been used for consecutive administrations [16] or alternatively by AAV capsid re-engineering [17]. Another issue relates to the cytopathogenicity of some viral vectors. For instance, alphaviruses cause a shut-down of host cell protein synthesis and induce apoptosis in infected mammalian cells leading to reduced therapeutic efficacy, which has been addressed by engineering non-cytopathogenic mutant vectors [18]. In the context of applying HIV-1 vectors for anti-HIV shRNA and miRNA delivery, the titers might be severely impaired due to the potential targeting of HIV-derived sequences resulting in a compromised therapeutic efficacy [19]. To address the problem, shRNAs can be produced to saturate the RNAi pathway or siRNAs/shRNAs targeting Dicer or Drosha can be applied. Alternatively, shRNA sequences deleted from the lentivirus vector can be selected, a human codon-optimized HIV-1 gag-pol vector can prevent shRNA targeting, or other types of lentiviruses such as HIV-1, simian immunodeficiency virus (SIV), feline immunodeficiency virus (FIV), bovine immunodeficiency virus (BIV) or equine infectious anemia virus (EIAV) can be utilized.

## 3. The Potential for Antiviral Drugs

RNAi-based gene silencing has been applied for Ad vectors targeting the mosquito-borne flavivirus Tembusu virus (TMUV) E and NS5 genes resulting in efficient down-regulation of TMUV RNA replication and virus production in Vero cells lasting for at least 96 h [20]. Ad-based efficient silencing of HBV replication was achieved by the delivery of artificial antiviral primary miRNAs (pri-miRNAs) [21], which was further improved to 94% inhibition in mice after an insertion of the liver-specific murine transthyretin (MTTR) promoter into the Ad vector [22]. Moreover, in the Special Issue, van den Berg and co-workers review RNAi-based gene silencing describing the RNAi pathway, RNAi activators, the design or synthetic siRNAs and chemical modifications to tackle HBV replication [7]. Furthermore, the potential of RNAi-based targeting of HBV covalently closed circular DNA (cccDNA) with various nonviral and viral vectors to suppress HBV replication is discussed. In the context of viral vector-based delivery, the transduction of liver cells is essential for HBV silencing. Ad vectors generally provide short-term expression and lentiviruses have demonstrated a low efficiency of hepatocyte transduction and issues related to oncogene activation after chromosomal integration have occurred. In contrast, AAV has emerged as a good candidate, especially the hepatotropic AAV8 serotype [23]. Self-complementary AAVs (scAAVs) provide faster transgene expression and have been shown to be suitable for the delivery of anti-HBV RNAi expression cassettes. The delivery of a single dose of 1 × 10^11^ scAAV8-anti-HBV copies inhibited HBV replication in transgenic mice for at least 10 months [24].

In another approach in the Special Issue, Eldabawy and co-workers engineered a novel JFH1-based HCV subgenomic replicon double reporter cell line for antiviral drug testing [8]. It allowed the rapid quantification of cell growth, viability, and HCV RNA replication. Application of lentivirus vectors expressing single, double or triple cassettes containing shRNAs targeting conserved HCV sequences and cellular factors involved in HCV replication resulted in reduced HCV replication. Stronger antiviral activity was observed for shRNAs targeting HCV sequences. In the case of anti-rotavirus drugs, miR-7 was shown to inhibit rotavirus replication by down-regulation of the expression of rotavirus nonstructural protein 5 (NSP5) [6]. Furthermore, the antiviral effect of miR-7 was demonstrated in a diarrhea suckling mouse model leading to the inhibition of rotavirus replication.

Related to self-replicating RNA viruses, six tandem targets for the neuron-specific miR124 sequences were introduced at the junction between the nonstructural protein genes nsP3 and nsP4 of the oncolytic replication-competent Semliki Forest virus (SFV) SFV4 vector [25]. BALB/c mice were intraperitoneally injected with SFV4-miRT124, which resulted in attenuated spread in the CNS and increased oncolytic activity in mouse CT-2A astrocytoma cells and human glioblastoma cell lines [26]. Additionally, SFV4-miRT124 was amplified in tumors and significantly inhibited tumor growth after a single intraperitoneal injection of C57BL/6 mice with orthotopic CT-2A gliomas. Moreover, miR124, miR125 and miR134 were introduced into the SFV4 vector, which prolonged survival and provided a cure in four out of eight A7J mice implanted with NXS2 neuroblastoma xenografts [27].

The current COVID-19 pandemic has also accelerated investigations into the potential application of RNAi strategies for antiviral therapy. Previously, four miRNAs and five siRNAs have been designed by computational calculations to target the ORF1ab region of the Middle East Respiratory Syndrome-Coronavirus (MERS-CoV) [28], providing the means for the potential inhibition of MERS-CoV replication although no wet-lab experiments have been carried out yet. However, the potential of the RNAi-based inhibition of coronavirus replication was demonstrated for porcine deltacoronavirus (PDCoV) by targeting the PDCoV M and N genes with plasmid-based shRNAs [29]. Plasmid transfection of swine testicular (ST) cells with M-shRNAs reduced PDCoV titers by 13.2-fold and viral RNA by 45.8%. Similarly, N-shRNA treatment resulted in a 32.4-fold decrease in titers and 56.1% reduction in RNA levels. Related to COVID-19, nine potential siRNAs targeting SARS-CoV-2 ORF1ab, ORF3a, S, M and N have been designed, ready for evaluation in cell lines and animal models [30]. Moreover, the 5’ end untranslated region (UTR) has been indicated as a target for noncoding RNAs and miRNA binding sites have been identified as suitable for the design of RNA-based drugs against SARS-CoV-2 [31].

## 4. Conclusions and Future Aspects

As can be read in the Special Issue on RNAi Interference for Antiviral Therapy and in the accumulated literature on the topic, RNAi has already been frequently used to target viral diseases. A number of examples have been presented on siRNA, shRNA and miRNA approaches leading to reduced viral titers and viral RNA levels. Specifically, it has been demonstrated for miRNAs targeting rotaviruses [6], for RNAi cassettes targeting HBV [7,24] and not the least the potential of developing RNAi-based antivirals for COVID-19 [30,31]. Interestingly, combinatorial lentivirus vectors expressing anti-HCV shRNAs have been engineered for the inhibition of HCV replication [8].

In general, RNAi-based therapeutics have strongly relied on nonviral delivery rather than applying viral vectors. Although delivery and stability issues have negatively affected the therapeutic efficacy, modifications of the 5’ 7-methylguanosine triphosphate (m7 G) cap structure, the poly (A) tail, the 5’ and 3‘end UTRs, and nanoparticle formulations based on liposomes, polymers, and hybrid lipid–polymers have provided substantial improvements. In this context, although not an antiviral drug, the RNAi-based ONPATTRO^TM^ (patisiran) was approved in August 2018 by the FDA for the treatment of polyneuropathy of hereditary transthyretin-mediated amyloidosis (hATTR) in adults [32].

In the context of viral vector-based RNAi therapy, the superior delivery and the unmatched expression levels are features making viral vectors superior. Particularly, self-replicating RNA viruses, which can be delivered as replication-deficient or -proficient particles, RNA replicons or DNA replicon plasmids, generate up to 200,000-fold RNA amplification in the cytoplasm of infected cells, resulting in unprecedented transgene expression levels. Naturally, administration of viral particles always needs to be applied under the highest possible safety conditions, which has triggered extensive research and the development of safer viral vectors. However, other issues of concern for viral vector-based RNAi therapy have been off-target effects of siRNA, which can lead to altered expression profiles of nontargeted transcripts [33] and the inhibition of protein translation resulting in nonspecific degradation [34]. Moreover, application of lentiviral vectors for RNAi-based HIV replication can lead to self-targeting, which might severely compromise the therapeutic efficacy as described above [19].

To end on a positive note, viral vector-based RNAi therapy for antiviral drugs has definitely showed some genuine promise as can be seen from the reviews and research articles in the Special Issue on RNAi Interference for Antiviral Therapy. The current COVID-19 pandemic has further accelerated antiviral drug development, which by taking the approach of “leaving no stone unturned” has also encouraged approaches of viral vector-based RNAi therapy against SARS-CoV-2. So, to answer the question of whether viral vectors are any good for RNAi antiviral therapy, we definitely are moving in that direction although we are not there, yet.
